# Involvement of TRPM7 in Alcohol-Induced Damage of the Blood–Brain Barrier in the Presence of HIV Viral Proteins

**DOI:** 10.3390/ijms24031910

**Published:** 2023-01-18

**Authors:** Michelle L. Mack, Wenfei Huang, Sulie L. Chang

**Affiliations:** 1 Institute of NeuroImmune Pharmacology, Seton Hall University, South Orange, NJ 07079, USA; 2 Department of Biological Sciences, Seton Hall University, South Orange, NJ 07079, USA

**Keywords:** ethanol, transient receptor potential melastatin 7, blood–brain barrier, brain microvascular endothelial cells, HIV-1, HIV-1 transgenic rat, HIV-1 envelope glycoprotein 120, TRPM7 antagonists

## Abstract

Ethanol (EtOH) exerts its effects through various protein targets, including transient receptor potential melastatin 7 (TRPM7) channels, which play an essential role in cellular homeostasis. We demonstrated that TRPM7 is expressed in rat brain microvascular endothelial cells (rBMVECs), the major cellular component of the blood–brain barrier (BBB). Heavy alcohol drinking is often associated with HIV infection, however mechanisms underlying alcohol-induced BBB damage and HIV proteins, are not fully understood. We utilized the HIV-1 transgenic (HIV-1Tg) rat to mimic HIV-1 patients on combination anti-retroviral therapy (cART) and demonstrated TRPM7 expression in rBMVECs wass lower in adolescent HIV-1Tg rats compared to control animals, however control and HIV-1Tg rats expressed similar levels at 9 weeks, indicating persistent presence of HIV-1 proteins delayed TRPM7 expression. Binge exposure to EtOH (binge EtOH) decreased TRPM7 expression in control rBMVECs in a concentration-dependent manner, and abolished TRPM7 expression in HIV-1Tg rats. In human BMVECs (hBMVECs), TRPM7 expression was downregulated after treatment with EtOH, HIV-1 proteins, and in combination. Next, we constructed in vitro BBB models using BMVECs and found TRPM7 antagonists enhanced EtOH-mediated BBB integrity changes. Our study demonstrated alcohol decreased TRPM7 expression, whereby TRPM7 could be involved in the mechanisms underlying BBB alcohol-induced damage in HIV-1 patients on cART.

## 1. Introduction

A HIV-1 transgenic (HIV-1Tg) rat model, developed by Reid et al. [1], contains a *gag*- and *pol*-deleted HIV-1 viral genome under the control of the LTR viral promoter, and persistently expresses 7 of the 9 HIV-1 genes. This rodent model has been well-characterized to mimic people living with HIV (PLWH) on combination anti-retroviral therapy (cART), in which viral replication is inhibited, but most viral proteins are still expressed [1,2,3,4]. We pioneered the use of this model to study the impact of the persistent presence of HIV-1 viral proteins at the molecular and behavioral levels [5,6] and have demonstrated that HIV-1Tg rats respond to addictive substances, including alcohol, more than the F344 control animals. 

Alcoholic beverages contain different percentages of ethanol (EtOH) termed alcohol-by-volume or ABV. Using a rodent model, we previously demonstrated that the physiological and behavioral effects of binge exposure to EtOH (binge EtOH) are ABV-dependent [7,8]. EtOH exerts its physiological effects through a variety of protein targets, including transient receptor potential (TRP) channels [9,10,11,12,13,14,15]. 

TRP channels are a group of cell membrane ion channels [16]. These channels comprise a large and functionally diverse superfamily of cell membrane cation channel proteins that are expressed in many cell types, and contain a large cytosolic N-terminal domain with unknown functions, a transmembrane domain for agonist binding and ion passage, and a cytosolic C-terminal domain for temperature sensing [17]. TRP channel proteins are generally described as the vanguard of our sensory system because they respond to a variety of intra-and intercellular stimuli [18,19], including temperature change [20,21] and noxious and immunologic challenges [18,19,22]. Seven subfamilies are categorized based on their specific agonists: ankyrin (TRPA), canonical (TRPC), melastatin (TRPM), mucolipin (TRPML), NOMPC (TRPN), polycystin (TRPP), and vaniloid (TRPV). The sequence identity of these subfamilies is as low as 20% [23,24]. Among the TRPM subfamily, TRPM7 is distinctive from other TRP channels because of its fusion and kinase domains. TRPM7 controls Ca^2+^ and Mg^2+^ homeostasis, with its activity being regulated by intracellular Mg^2+^ [25,26,27,28,29,30,31]. There exists a growing body of literature indicating the involvement of TRPM7 in key cellular processes including cell proliferation and survival [32,33,34,35,36,37,38,39,40,41,42,43,44], suggesting TRPM7 is required for proper cellular communication within the tissue microenvironment [35,37,40,43,45,46,47,48]. Deregulated expression or function of TRPM7 contributes to the pathophysiology of many prominent diseases such as cancer [49,50,51], ischemic stroke [52] and cardiovascular disease [53,54,55,56]. 

Various TRP channels are expressed in numerous cell types, including brain microvascular endothelial cells (BMVECs), the major cellular component of the blood–brain barrier (BBB) [57,58]. The BBB is a highly selective permeable barrier that separates circulating blood from the extracellular fluid in the brain, with its integrity being essential for maintaining brain health. BMVECs are key components of the BBB and contain tight junction (TJ) complexes consisting of occludin, claudins, and zonula occludens (ZO) that restrict paracellular passage across the BBB, ensuring its structural and functional integrity [59,60]. Death (apoptosis) of BMVECs is a biomarker of BBB damage [61,62], and many pathological conditions, including HIV infection [63], traumatic brain injury [64], and alcohol abuse [65,66], can damage BMVECs and increase permeability of the BBB. Binge drinking is known to cause gut leaking resulting in elevated peripheral blood endotoxin levels. These effects include dysregulation of neuroinflammation [63,65,66], leading to EtOH-induced apoptosis of BMVECs and, ultimately, damage to the BBB [67]. In PLWH, there are significant alterations in both BBB integrity and tight junction protein expression [68], such as elevated markers for vascular permeability [69,70,71] and higher expression of adhesion molecules, such as platelet endothelial cell adhesion molecule [PECAM] [72]. Heavy alcohol drinking is often associated with HIV infection or head trauma [73,74,75]. Compared to the broader community, the rate of heavy alcohol drinking is twice as high in PLWH [75,76]. Additionally, people in general have a 12–18% lifetime history of alcohol use disorder (AUD) in contrast to PLWH being that of 55% [75,77,78]. While studies have demonstrated that damage to the BBB can be caused by alcohol-induced apoptosis of BMVECs [67], the mechanisms underlying alcohol-induced BBB damage in the presence of HIV viral proteins have not been fully delineated and it is expected that alcohol damage to the BBB will be a comorbidity with PLWH. Thus, we utilized the HIV-1 transgenic rat as our study model to mimic PLWH and BBB dysfunction to interrogate the molecular mechanisms underlying vascular endothelial cell dysregulation and HIV viral proteins.

Using real-time PCR, we previously detected significantly higher expression of TRPM7 in primary mouse BMVECs [79]. We then demonstrated that TRPM7 expression was lower in HIV-1Tg rats at 3–4 weeks of age compared to that in F344 control rats, whereas both F344 and HIV-1Tg rats expressed similar TRPM7 levels at 9 weeks or older, indicating that the persistent presence of HIV-1 viral proteins delayed TRPM7 expression in adolescents. Repeated binge exposure to EtOH decreased TRPM7 expression in BMVECs of F344 rats in a concentration-dependent manner, while it completely abolished expression of TRPM7 in BMVECs of the HIV-1Tg rats. Using TRPM7 antagonists (CyPPA, SKA-31 and NS 8593), in both rodent and human in vitro BBB models, we demonstrated the involvement of TRPM7 in EtOH-mediated changes of the BBB permeability. Treatment with EtOH downregulated TRPM7 expression and increased permeability of the BBB in vitro. These data revealed that blocking TRPM7 disrupted the integrity of the BBB, and that TRPM7 was critical in the function of the vascular endothelial barrier. 

## 2. Results

### 2.1. Members of 6 TRP Subfamilies Are Expressed in Mouse BMVECs

To provide rationale for TRPM7 investigation, data was taken [79] and tabulated to show expression levels of 29 mammalian TRP genes in mouse BMVECs (mBMVECs). We used a custom-made PCR array, and found that TRPM7 was highly expressed in mBMVECs compared to most other TRP channels. Expression levels (2^−ΔΔCt^ values) of TRP genes relative to those of control housekeeping genes are listed (Table 1). Six TRP genes with high expression levels included Trpc1, Trpm4, Trpm7, Trpml1, Trpml2, and Trpp2, genes with moderate expression were Trpc2, Trpc5, Trpc7, Trpm1, Trpm2, Trpm3, Trpm5, Trpv2, Trpv4, Trpml3, and Trpp5, while other TRP gene expression was undetected. (n = 3 technical replicates).

### 2.2. TRP Channel Protein Expression in Mouse BMVECs

**Figure 1:**
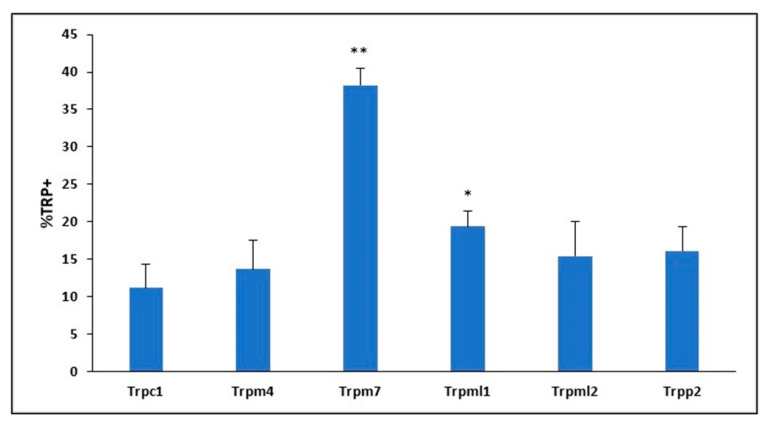
TRP protein channel expression in mouse BMVECs. * *p* < 0.05, ** *p* < 0.01.

To further explore TRP channels with high RNA expression, we proceeded to interrogate protein expression by flow cytometry. Analysis revealed 38% TRPM7+ protein expression in mouse BMVECs; whereas all other TRP channels screened exhibited less than 20% protein expression within the mBMVEC population. Specifically, 11% TRPC1+, 14% TRPM4+, 19% TRPML1+, 15% TRPML2+ and 16% TRPP2+ (Figure 1). TRPM7 was chosen to be studied further due to the high protein expression in mBMVECs as compared to other TRP proteins examined. 

### 2.3. Ethanol’s Effects on TRPM7 Protein Expression in Mouse BMVECs

**Figure 2:**
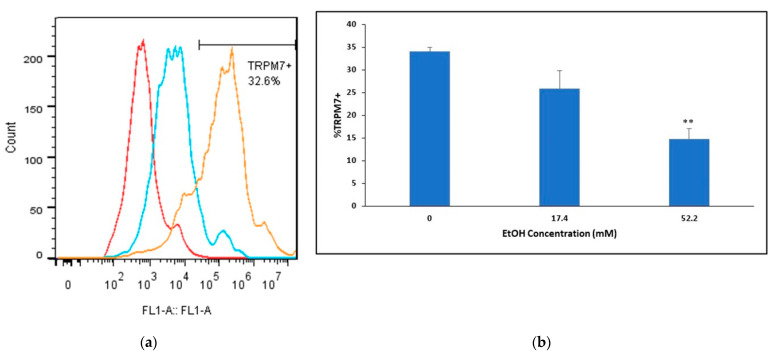
TRPM7 expression in mouse BMVECs following EtOH treatments: (**a**) Representative histograms of intracellular TRPM7. Mouse BMVECs were stained with primary TRPM7 Ab (orange), isotype control (blue), or unstained (red); (**b**) Mouse BMVECs treated with 17.4 mM EtOH, 52.2 mM EtOH, or no EtOH (control) for 24 h. Cells were stained for TRPM7 expression. ** *p* < 0.01.

We confirmed mouse TRPM7 basal protein expression and examined whether TRPM7 expression levels were modulated by ethanol (EtOH). Flow cytometry analysis determined a 34% TRPM7+ population in mBMVEC (passage 6) primary cell cultures (Figure 2a). EtOH’s modulation of TRPM7 protein levels following 24 h treatment with either 17.4 mM or 52.2 mM by flow cytometry analysis revealed TRPM7 expression is downregulated in a concentration-dependent manner. High-dose (52.2 mM) EtOH treatment significantly downregulated TRPM7 expression by greater than 2-fold, whereas 17.4 mM EtOH treatment for 24 h decreased TRPM7 expression by 24% (Figure 2b).

### 2.4. Ethanol’s Modulation on the Differential Expression of TRPM7 in BMVECs from F344 and HIV-1Tg Rats 

**Figure 3:**
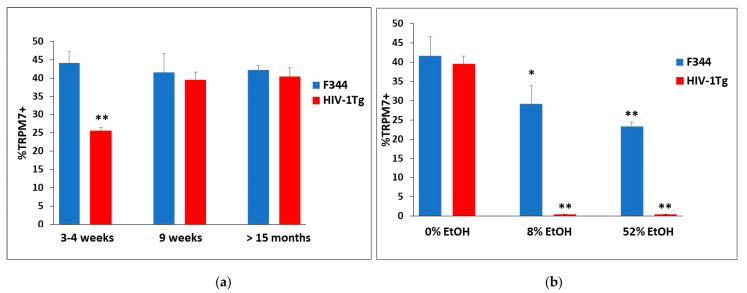
Flow cytometry analysis of TRPM7 expression from HIV-1Tg rBMVECs compared to F344 age-matched control rat BMVECs: (**a**) Differential expression of TRPM7 protein between HIV-1Tg rats and F344 control animals at different ages (3–4 weeks, 9 weeks and >15 months); (**b**) Nine wk old F344 and HIV-1Tg rats were binge treated with either 0%, 8%, or 52% EtOH for 4 wks, and harvested rat BMVECs were stained for intracellular TRPM7 protein. Each sample for flow cyotmetric analysis consisted of 3 rat brains and was repeated in 3 independent experiments, run in triplicate. * *p* < 0.05, ** *p* < 0.01.

To examine TRPM7 differential expression between HIV-1Tg rats and age-matched F344 controls, with and without EtOH binge treatment, we analyzed rBMVECs by flow cytometry. TRPM7 expression was lower in HIV-1Tg rats at 3–4 wks of age (25.6% TRPM7+) compared to that in the F344 control rats (44.1%), whereas both F344 and HIV-1Tg rats expressed similar TRPM7 levels (42.2% and 40.4.6%, respectively) at 15 months or older (Figure 3a). Nine-week-old HIV-1Tg and F344 rats were randomly assigned to receive either saline or EtOH (2 g/kg/d, 3d, i.g.) at either 8% or 52% (*v*/*v*) solution for 4 weeks. Twenty-four hours after the last treatment with EtOH or saline, the brains of HIV-1Tg and F344 rats at the age of 9 wks were collected and used to isolate BMVECs, which were analyzed by flow cytometry for expression of TRPM7 protein. Binge exposure to an 8% EtOH (*v*/*v*) solution in F344 rats resulted in 29.2% TRPM7+ expression, whereas F344 control animals that received a 52% (*v*/*v*) binge exhibited 23.3% TRPM7 expression. Binge exposure to EtOH for 4 weeks decreased TRPM7 expression in the F344 rat BMVEC (rBMVECs) in a concentration-dependent manner (Figure 3b); however, HIV-1Tg rats exhibited complete downregulation of TRPM7 to an undetectable level when given either 8% or 52% EtOH.

### 2.5. Involvement of TRPM7 in Ethanol-Mediated BBB Permeability Changes

**Figure 4:**
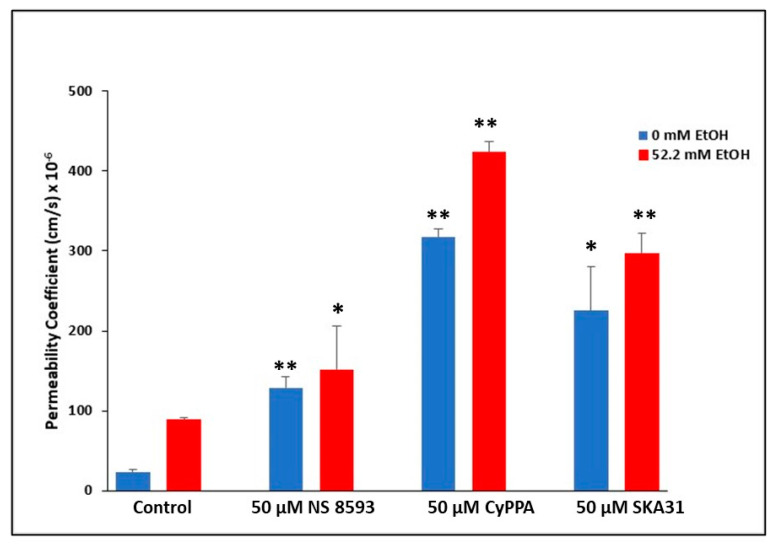
Rat BMVEC in vitro BBB model. Passage 5 isolated rBMVECs were seeded into Corning transwell plate inserts and grown for three days, to allow a barrier to form as confirmed by Transendothelial Electrical Resistance (TEER) measurements. Media only was added to wells to account for background. BMVECs were treated with either 0 or 52.2 mM EtOH, as well as TRPM7 antagonists, NS 8593, CyPPA, or SKA31 in both 0 and 52.2 mM EtOH treatment conditions. Ten μg/mL of Sodium Fluorescein (NaF) was added to each insert for permeability assessment prior to assay incubation at 37 °C for 24 h. Permeability coefficient was calculated, along with *p*-value by Student’s *t* test. * *p* < 0.05, ** *p* < 0.01.

In line with EtOH’s downregulation of TRPM7 in rodent BMVECs at both the protein and mRNA levels [79], we proceeded to examine the modulatory effects of EtOH on BBB permeability mediated by TRPM7. BBB integrity was assessed using an in vitro rat BMVEC model, treated with either 0 or 52.2 mM EtOH, as well as 50 μM TRPM7 antagonists, NS 8593, CyPPA, or SKA31 in both 0 and 52.2 mM EtOH treatment conditions for 24 h. Sodium Flourescein (NaF) was used as a tracer, and added to inserts, to detect permeability changes within the endothelial cell barrier. Treatment with 52.2 mM EtOH for 24 h significantly increased permeability (*p* < 0.01). All three TRPM7 blockers increased BBB permeability significantly (*p* < 0.05) compared to control (no TRPM7 blocker), both with (52.2 mM EtOH) and without (0 mM) ethanol treatments (Figure 4). TRPM7 inhibitor, CyPPA, significantly increased BBB permeability when treated with 52.2 mM EtOH compared to 0 mM EtOH CyPPA treated rBMVECs over 24 h (*p* < 0.01).

### 2.6. Analysis of TRPM7 Expression in Human BMVECs after Ethanol and HIV-1 gp120 Combination Treatment

**Figure 5:**
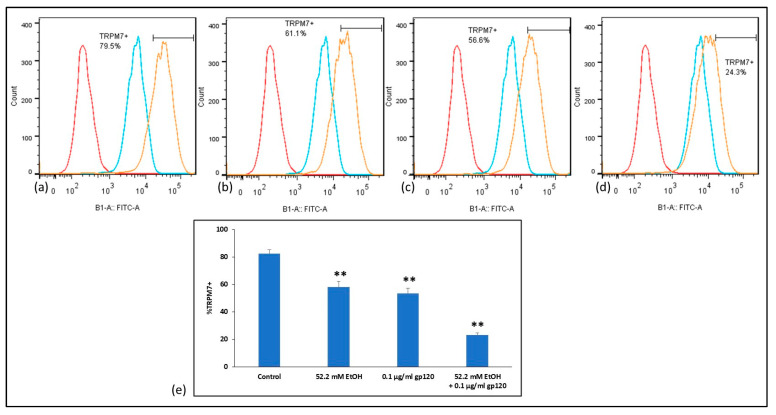
Flow cytometry analysis of TRPM7 expression in human BMVECs: (**a**–**d**) Representative histograms of intracellular TRPM7. Human BMVECs were stained with primary TRPM7 Ab (orange), isotype control (blue), or unstained (red); (**e**) Human BMVECs treated with 52.2 mM EtOH, HIV-1 gp120, and combination treatment of 52.2 mM EtOH plus gp120 for 24 h. Cells were stained for TRPM7 expression. ** *p* < 0.01.

In order to provide a translational value in clinically caring for PLWH with alcohol abuse, we proceeded to investigate the modulation of TRPM7 protein expression after EtOH and HIV viral protein treatments in human primary cultures. Flow cytometry analysis confirmed 80% TRPM7+ population in passage 8 human BMVEC primary cell cultures (Figure 5a,e). Modulation of TRPM7 protein levels following 24 h treatment with 52.2 mM EtOH by flow cytometry analysis revealed TRPM7 expression is significantly downregulated to 61% (Figure 5b,e); similarly, 0.1 µg/mL HIV-1 gp120 treatment downregulated TRPM7 expression to 57% (Figure 5c,e). Combination treatment of 52.2 mM EtOH and gp120 for 24 h significantly downregulated TRPM7 expression by greater than 3-fold (Figure 5d,e). Flow cytometry analysis was repeated in 3 independent experiments, run in triplicate. 

### 2.7. Ethanol and HIV-1 Viral Protein gp120 Effects on Tight Junction Complexes

**Figure 6:**
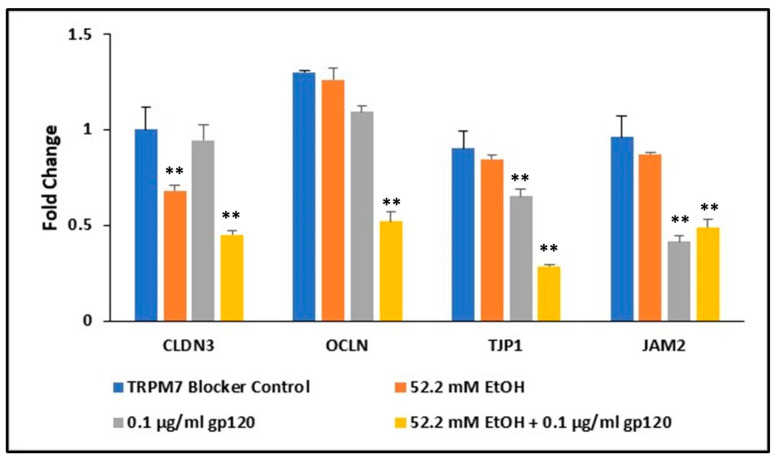
Fold change in various tight junction complexes gene expression in human BMVECs following 24 h treatment. ** *p* < 0.01.

In line with tight junction proteins ensuring BBB integrity, we interrogated the downregulation of TRPM7 induced by HIV proteins and EtOH leading to the reduced expression of tight junction proteins in primary human BMVECs. Alterations in tight junction gene expression by EtOH and HIV-1 viral protein gp120 was confirmed by qRT-PCR. Gene expression in human BMVECs was assessed after a 24 h treatment with TRPM7 blocker control (50 µM CyPPA), 52.2 mM EtOH, 0.1 µg/mL HIV-1 gp120, and a combination of 52.2 mM EtOH plus gp120. Monotherapies and combinations were also treated with TRPM7 antagonist, CyPPA. Claudin-3 (CLDN3) expression was decreased by more than 30% following treatment with 52.2 mM EtOH, whereas both tight junction protein-1 (TJP1) and junctional adhesion molecule-2 (JAM2) were reduced following HIV-1 gp120 treatment, 35% and 60% respectively (Figure 6). All four tight junction complexes were significantly affected following a 24 h combination treatment with 52.2 mM EtOH and gp120. CLDN3, JAM2, and occludin (OCLN) were significantly downregulated by approximately 50%, and TJP1 by over 70%. 

### 2.8. Involvement of TRPM7 in Alcohol Induced Damage of the BBB in the Presence of HIV Viral Protein gp120

**Figure 7:**
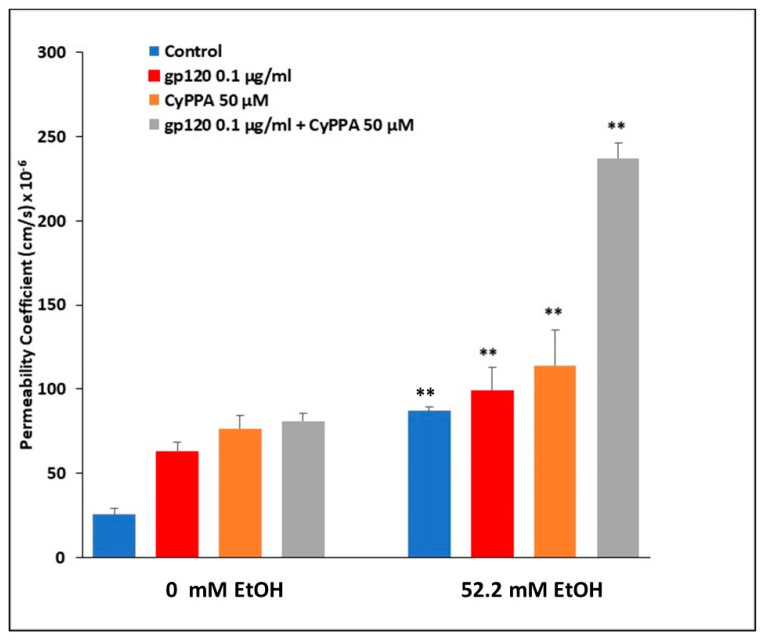
Human BMVEC in vitro BBB model. Cells were seeded into Corning transwell plate inserts and grown for three days, to allow a barrier to form as confirmed by TEER measurements. Media only was added to wells to account for background. BMVECs were treated with either 0 or 52.2 mM EtOH, 50 µM TRPM7 antagonist (CyPPA), 0.1 µg/mL HIV-1 gp120, alone and in combination treatment conditions. Ten μg/mL of Sodium Fluorescein (NaF) was added to each insert for permeability assessment prior to assay incubation at 37 °C for 24 h. Permeability coefficient was calculated, along with *p*-value by Student’s *t* test. ** *p* < 0.01.

Centered upon HIV-1 viral protein gp120 and ethanol’s synergistic downregulation of TRPM7 in human BMVECs, we examined the combined effects of EtOH and gp120 on BBB permeability mediated by TRPM7. BBB integrity was assessed using an in vitro human BMVEC model, treated with either EtOH (0 or 52.2 mM), 50 µM TRPM7 antagonist, CyPPA, or 0.1 µg/mL HIV-1 gp120, administered both as monotherapies and in combinations for 24 h. Sodium Flourescein (NaF) was used as a tracer, and added to inserts, to detect permeability changes within the endothelial cell barrier. Treatment with 52.2 mM EtOH for 24 h significantly increased permeability (Figure 7). CyPPA, HIV-1 gp120 and the combination treatment of CyPPA plus gp120 increased BBB permeability significantly (*p* < 0.01) in the presence of 52.2 mM EtOH compared to control (0 mM EtOH).

## 3. Discussion

Many rodent models have been developed to study HIV-associated disorders [1,80,81,82,83,84,85,86,87,88,89,90,91,92]. The non-infectious HIV-1 transgenic (HIV-1Tg) rat, developed by Reid et al. [1], exhibits various manifestations of human HIV infection in the absence of viral replication. This rodent model has been well-characterized to mimic persons living with HIV (PLWH) on combination anti-retroviral therapy (cART), in which viral replication is inhibited, but most viral proteins are still expressed [1,2,3,4]. We pioneered the use of this model to study the impact of the persistent presence of HIV-1 viral proteins at the molecular and behavioral levels [5,6] and have demonstrated that HIV-1Tg rats respond to addictive substances, including alcohol, more than the F344 control animals. Thus the HIV-1Tg that was generated has been characterized to be the ideal model to mimic PLWH given cART [4]. 

Ethanol (EtOH) exerts its numerous effects through a variety of protein targets [13,65,93,94,95], particularly ion channels [96], such as EtOH targeting transient receptor potential (TRP) channels [13]; however, reports on the effects of EtOH-TRP binding are contradictory. TRPV1 and TRPA1 channels are activated by EtOH [11,14,15], whereas TRPM8 activity is inhibited by EtOH at high concentrations [9], and TRPV1 has been shown to play a role in taste-induced alcohol avoidance [12] and alcohol-induced intoxication [10] in TRPV1 knockout (KO) mice. 

Using real-time PCR, we have previously reported that expression of TRPM7 is much more abundant than the other TRP channels in brain microvascular endothelial cells (BMVECs) and treatment with EtOH decreases TRPM7 over time [79]. Although TRPV1, TRPM8, and TRPA1 mediate various EtOH effects in animal models [10,12,15], the expression of these three TRP channels in BMVECs is not definitive. TRPM7, a melastatin subfamily closely related to TRPM8, is heavily expressed in BMVECs and affected by EtOH. TRPM7 is distinctive from other TRP channels because of its fusion and kinase domains that are permeable to both Ca^2+^ and Mg^2+^ [97]. Through the regulation of intracellular Mg^2+^ levels, TRPM7 has been shown to play an important role in the activation of immune responses [98]. Due to the high TRPM7 protein expression in BMVECs, we therefore decided to further investigate EtOH binding to TRPM7 at the molecular level and examine EtOH’s effects on immune responses in the presence of HIV-1 viral proteins using rodent and human BMVEC primary cultures.

In addition to TRPM7, we identified five other TRP channels with high expression detected by real-time PCR that have been implicated in BBB integrity, to include TRPC1, TRPP2, TRPM4, TRPML1 and TRPML2. Activation of TRPC1 and TRPP2 channels elevates cytosolic calcium levels and increases endothelial permeability, which could compromise the integrity of the BBB [57,58]. While TRPM4 regulates intracellular calcium overload and oscillation, as well as supporting immune cell migration [99]. It has been previously reported that TRPM4 is critical for oxidative stress enhanced endothelial cell migration [100]. Two members of the mucolipin family were also expressed in BMVECs. TRPML proteins are non-selective Ca^2+^ permeable intracellular channels located on the lysosomal membrane. This subfamily plays a role in both endolysosomal processes and endocytosis [101]. Although the precise role of TRPML channels has yet to be elucidated, this subfamily has been linked to neurodegenerative disorders due to oxidative stress and autophagy activation [102]. Protein expression of these five subfamily channels in mouse BMVECs was minimal and did not warrant further investigation.

Compared to the broader community, the rate of heavy alcohol drinking is twice as high in PLWH [75,76]. Additionally, people in general have a 12-18% lifetime history of AUD in contrast to PLWH being that of 55% [75,77,78]. Hence, there exists a serious clinical problem for alcohol addicted PLWH. We used the HIV-1 transgenic rat as our study model to mimic PLWH and BBB dysfunction to interrogate the molecular mechanisms underlying vascular endothelial cell dysregulation and HIV viral proteins. The HIV-1Tg rat mimics PLWH on cART, due to this animal having no viral replication but 7 of 9 viral proteins being persistently expressed [1,2,3,4]. Flow cytometric analysis revealed a differential expression of TRPM7 protein between HIV-1Tg rats and F344 control animals at different ages (4–5 weeks vs. 12–16 months). TRPM7 expression was lower in HIV-1Tg rats at 4–5 weeks of age compared to that in the F344 control rats, whereas both F344 and HIV-1Tg rats expressed similar TRPM7 levels at 12 months or older (Figure 3a). These data provide confirmation that the persistent presence of HIV-1 viral proteins delays TRPM7 expression in young HIV-1Tg rats.

A multiplicity of studies have shown that binge exposure to EtOH causes disruption of the BBB [67,103], wherein BMVECs are the key cellular component [57,58]. Both physiological and behavioral effects of binge exposure to EtOH (binge EtOH) in F344 rats have been shown to be alcohol-by-volume (ABV) dependent [7,8]. We demonstrated high levels of TRPM7 expression in BMVECs, and further showed that treatment with various concentrations of EtOH differentially modulated TRPM7 expression. Binge exposure to EtOH for 4 weeks decreased TRPM7 expression in BMVECs of the F344 rats in a concentration-dependent manner; however, HIV-1Tg rats exhibited complete downregulation of TRPM7 to an undetectable level when given either 8% or 52% EtOH (Figure 3b). These data showed that binge EtOH exposure completely blocks TRPM7 expression in HIV-1Tg rats, and we hypothesized this to be one of the mechanisms underlying damage to the BBB caused by repeated binge alcohol in PLWH who are on treatment with cART. 

While studies have demonstrated that damage to the BBB can be caused by alcohol-induced apoptosis of BMVECs [67], the mechanisms underlying alcohol-induced BBB damage in the presence of HIV viral proteins have not been fully delineated and are vastly understudied. However, it is expected that alcohol damage to the BBB to be a comorbidity with PLWH resulting in detrimental effects to the integrity of the BBB, including increases in leukocyte endothelial adhesion (LEA). Therefore, the BBB plays an important role in immune privilege of the central nervous system (CNS), with disruption of the BBB being detrimental since various pathogens may enter the brain compartment once the BBB integrity has been compromised. Functional studies of specific genes, such as TRPM7, in primary human cells are critical to elucidate the dysregulation of the BBB in PLWH. Flow cytometry results revealed high TRPM7 expression in human BMVECs, and the downregulation of TRPM7 is modulated by EtOH and HIV viral proteins in a synergistic manner (Figure 5).

Prior to investigating BBB model systems, we initially explored TRPM7 expression following treatment with all three antagonists (CyPPA, SKA-31 and NS 85093), utilizing flow cytometry and qRT-PCR techniques. We demonstrated at both the protein and mRNA levels, that TRPM7 expression was significantly downregulated following 24 h antagonist treatments in rodent and human primary cultures. Based on the downregulation of TRPM7, we then proceeded to explore BBB permeability changes mediated by TRPM7. Utilizing both rodent and human in vitro BMVEC BBB models, the downregulation of TRPM7 expression, by way of TRPM7 blockers, coupled with the presence of HIV viral proteins, significantly enhanced EtOH-mediated changes of the BBB (Figure 4 and Figure 7). 

In both BMVEC barrier models, there was significantly higher dysregulation of the endothelial integrity with CyPPA compared to other TRPM7 antagonists. CyPPA and SKA-31 both block TRPM7 and trigger Ca^2+^ activated potassium channels (K_Ca_) that selectively transport K^+^ ions across cellular membranes. The cytosolic Ca^2+^ concentration is then increased [104], resulting in apoptotic Ca^2+^ signaling [105]. These channels can be classified into three subtypes: small (SK), intermediate (IK) and big conductance (BK) channels. CyPPA is an activator of small conductance K_Ca_ channels that displays selectivity for SK2 and SK3 channels and does not target SK1 or IK channels [106]. SKA-31 activates small and intermediate K_Ca_ channels [107]; whereas NS 8593 both blocks TRPM7 channels and inhibits the modulation of all small K_Ca_ channels, having no effect on any other K_Ca_ channels [108]. Taken together, the increased dysregulation of the BBB integrity from CyPPA may be attributed to the pharmacological overlap of blocking TRPM7 and activating K_Ca_ channels, in addition to the selectivity for SK2 and SK3 channels present in primary endothelial cultures and should be further explored.

To investigate the underlying disruption of the endothelial barrier integrity, we confirmed a significant decrease in the expression of endothelial cell tight junction complexes, Claudin-3 (CLDN3), occludin (OCLN), junctional adhesion molecule-2 (JAM-2) and tight junction protein-1 (TJP1), also known as zonula occludens (ZO-1), following treatment with HIV viral proteins and EtOH in human BMVECs (Figure 6). This decreased expression could inhibit the restrictive control of the tight junction complexes, allowing paracellular passage across the BBB. Our data have shown that the combination of TRPM7 antagonists, EtOH and HIV viral proteins worked in a collaborative manner to further disrupt BBB permeability (Figure 7). This study provides evidence that alcohol decreases TRPM7 expression, whereby TRPM7 is one of the mechanisms underlying alcohol induced damage at the BBB in HIV-1 patients on cART treatment.

## 4. Materials and Methods

### 4.1. Animals

Male HIV-1Tg and F344 rats at 3–4 weeks of age were purchased from Harlan Inc. (Indianapolis, IN, USA). All rats were housed in ventilated cages (Animal Care Systems, Inc., Littleton, CO, USA) and maintained in a temperature- and humidity-controlled environment with a 12 h light/dark cycle and ad libitum access to standard rat chow and water throughout the duration of the study. The rats were monitored daily, and body weights recorded. This research has been approved by the Institutional Animal Care and Use Committee at Seton Hall University, South Orange, NJ, USA.

### 4.2. EtOH Administration 

To minimize stress associated with intragavage (i.g.) feeding, the animals were conditioned to the procedure for 2 d using water (1.5 mL/kg/d). The animals were then randomly divided into 3 groups per strain and given either an 8% or 52% ethanol (EtOH) solution (2 g/kg/d, 3 d; n = 3), or water (0% control group, n = 2) by i.g. administration once a day for 4 weeks. This EtOH treatment regimen is to mimic repeated binge drinking of the adolescent, published previously [8,109].

### 4.3. Tissue Collection and BMVEC Isolation 

The rats were sacrificed at 24 h ± 1.5 h after the last treatment using a guillotine. After sacrifice, the brains were collected, weighed, and relative brain size determined by calculating the brain wt/body wt (br-wt) ratio for each rat. BMVECs were isolated from the combined tissue of each group (n = 3) using a modified version of the proprietary protocol provided by Cell Biologics (Chicago, IL, USA). The above procedure was repeated three times for a combined tissue and isolated BMVEC in each group of n = 9. 

### 4.4. Cell Culture

Isolated rat BMVECs were cultured with complete rat endothelial cell medium (M1266, Cell Biologics, Chicago, IL, USA), plus supplements (0.5 mL EGF, 0.5 mL VEGF, 5 mL antibiotic-antimycotic and 10 mL FBS). Primary mouse and human BMVECs were purchased from Cell Biologics (Chicago, IL, USA) and cultured with their respective complete endothelial cell medium and supplements. Human endothelial cell medium (H1168, Cell Biologics) was supplemented with 0.5 mL EGF, 0.5 mL VEGF, 0.5 mL FGF, 0.5 mL heparin, 0.5 mL hydrocortisone, 5 mL L-Glutamine, 5 mL antibiotic-antimycotic and 25 mL FBS. Mouse endothelial cell medium (M1168, Cell Biologics) was supplemented with 0.5 mL EGF, 0.5 mL VEGF, 0.5 mL ECGS, 0.5 mL heparin, 0.5 mL hydrocortisone, 5 mL antibiotic-antimycotic and 25 mL FBS. All primary endothelial cells were grown in a 37 °C humidified incubator with 5% CO_2_, in gelatin coated flasks to 80% confluency, with morphology verified by microscopy daily. Cell culture media was aspirated, cells washed twice with PBS, and detached using trypsin/edta. Trypsin was then neutralized by the addition of cell culture media to the flask. Cell suspensions were filtered through a 100 μm sterile cell strainer (Fisher Scientific, Nazareth, PA, USA) and centrifuged. Supernatants were then aspirated, and cell pellets resuspended in fresh media, prior to determining viability by trypan blue using the Countess automated cell counter (Invitrogen, Thermo Fisher Scientific, Waltham, MA, USA). Throughout this study, cells were split at a ratio of 1:3.

### 4.5. Flow Cytometry

BMVECs (10^6^ cells/sample) were analyzed by flow cytometry using ZO-1 endothelial marker (Cat#617300, Life Technologies, Thermo Fisher Scientific, Waltham, MA, USA) for rodents, and CD31 (Cat#561564, BD Biosciences, San Jose, CA, USA) for human endothelial cells. Mouse and human BMVECs purchased from Cell Biologics required treatment with various concentrations of EtOH and HIV-1 gp120 (NIH-ARP, 4961) for flow cytometry analysis, compared to rBMVECs where treatments occurred in vivo. Mouse BMVECs were blocked for 30 min at room temperature (RT) with mouse Fc Block (Cat#553141, BD Biosciences, San Jose, CA, USA) to prevent nonspecific staining. One microgram of Fc block was used per 10^6^ cells in 100 µL of staining buffer (PBS/1%BSA). To block non-specific staining for rBMVECs, 0.25 µg anti-rat CD32 (Cat#550271, BD Biosciences, San Jose, CA, USA) was used and cells were preincubated for 5 min at 4 °C; whereas hBMVECs were blocked in staining buffer (PBS/1%BSA) for 30 min at RT. BMVECs were pelleted by centrifugation (400× *g*) at 4 °C for 5 min, then fixed and permeabilized according to the manufacturer’s protocol (BD Biosciences, San Jose, CA, USA), followed by staining with intracellular TRPM7 antibody purchased from Abcam (Cat#ab85016, Abcam, Cambridge, UK) or TRP antibodies purchased from Alomone Labs (Cat#ACC-010, ACC-044, ACC-047, ACC-052, ACC-081, ACC-082, Alomone Labs, Jerusalem, Israel) in Perm/Wash™ solution at 4 °C for 30 min, protected from light. Following incubation, BMVECs were pelleted by centrifugation (400× *g*) at 4 °C for 5 min and washed twice with 1X Perm/Wash™ solution. BMVECs were then incubated at 4 °C for 30 min in the dark with a species appropriate fluorochrome-conjugated secondary anti-IgG antibody (Jackson, West Grove, PA, USA). BMVECs were once again pelleted by centrifugation and washed twice. Stained BMVECs were then re-suspended in staining buffer, in triplicate, and 10,000 events were acquired on the Accuri C6 (BD, Franklin Lakes, NJ, USA).

### 4.6. In Vitro BBB Permeability Assay

Using isolated BMVECs we constructed an in vitro endothelial barrier in Corning transwell plate inserts [insert area = 0.33 cm^2^; pore size = 0.4 μM] (Corning Life Sciences, Tewksbury, MA, USA). A modified previously published protocol was used [110,111]. Briefly, 1200 μL cell culture medium was added to each bottom well and 300 μL BMVEC cell suspension (5 × 10^4^ cells/insert) to the upper insert. Transwell plates were then incubated at 37 °C with 5% CO_2_. Formation of the endothelial barrier was monitored daily and formed within three days when the endothelial cells became confluent, as confirmed by daily Transendothelial Electrical Resistance (TEER) measurements. Various concentrations of EtOH were added to both the inserts and bottom of the wells, as well as cell culture incubator trays. HIV-1 viral protein, gp120 (NIH-ARP, 4961) and three different TRPM7 Channel Blockers, NS 8593, CyPPA and SKA-31 (Alomone Labs, Jerusalem, Israel) were added to inserts and bottom of the wells only. Ten μg/mL of sodium fluorescein (NaF) (Cat#F6377, Sigma-Aldrich, St. Louis, MO, USA) was utilized as a tracer and added to the upper insert to detect permeability changes. Plates were then incubated in a humidified cell culture incubator at 37 °C with 5% CO_2_ for 24 h. One hundred microliter samples from both the upper insert and the lower chamber were transferred to a clear 96-well plate. Fluorescence of NaF was measured using a Gemini EM microplate reader (Molecular Devices, Sunnyvale, CA, USA) with an excitation wavelength of 480 nm and an emission wavelength of 535 nm. The permeability was calculated as the amount of NaF passing across the endothelial layer in 24 h [112]. Permeability coefficient was measured and calculated based on previously published protocols [113]. Briefly, the Clearance (cm^3^) of tracer diffusing from the luminal (insert) to abluminal chamber was calculated by the following formula: Clearance (cm^3^) = [Concentration]_Abluminal_ × [Volume]_Abluminal_/[Concentration]_Luminal._ [Concentration]_Luminal_ is the initial tracer concentration within the insert loaded with tracer; [Concentration]_Abluminal_ is the tracer concentration in the abluminal chamber following treatment. During the experiment, the tracer volume diffused from luminal (insert) to abluminal increased linearly with time. Permeability-surface area product is abbreviated as PS. PS_time-plotted_ was calculated by Clearance (cm^3^) divided by time (s). PS_endothelial_ was calculated by 1/PS_endothelial_ = 1/PS_time-plotted_ − 1/PS_insert_. PS_endothelial_ divided by the surface area generated the permeability coefficient (cm/s).

### 4.7. RNA Isolation, Purification, Reverse Transcription, and Quantitative RT-PCR Array 

RNA extraction, reverse transcription, and qRT-PCR were performed following treatment with various concentrations of TRPM7 antagonists, EtOH, HIV-1 gp120 (NIH-ARP, 4961), and combination treatments. Total RNA was isolated from BMVEC cultures using the RNeasy Mini Kit (Qiagen, Germantown, MD, USA) according to the manufacturer’s protocol. The purity and quality of total RNA was determined using an ND-1000 spectrophotometer (NanoDrop Technologies, Inc., Wilmington, DE, USA). An equal amount of RNA from each sample (400 ng) was converted to cDNA using the RT2 First-Strand Kit (Qiagen, Germantown, MD, USA). A custom-designed RT-PCR array was used to determine mRNA expression. Primers of each gene were designed with Primer Express (v. 3.0) (Applied Biosystems, Carlsbad, CA, USA) and spanned at least one intron to avoid amplifying genomic DNA. Each pair of primers and their amplicon sequences were tested using the Basic Local Alignment Search Tool (BLAST; https://blast.ncbi.nlm.nih.gov/Blast.cgi, accessed on 3 May 2022) to ensure the specificity of the primers. Using the ABI Prism 7900HT Fast Detection System, cDNA samples were amplified in a 10-μL volume containing 5 μL of RT2 SYBR Green PCR Master Mix (Qiagen), primers for each gene (2.5 μL; final concentration 250 nM), and 2.5 μL of diluted cDNA in a 384-well plate (Thermofisher, Waltham, MA, USA). Target gene expression was normalized to that of the housekeeping gene. Gene expression was calculated using the 2^−∆∆Ct^ method. Melting curve analysis was applied to characterize the specificity of the amplifications.

### 4.8. Statistical Analysis 

Flow cytometry analysis was performed using FlowJo V10. All data are expressed as the mean ± standard deviation (S.D.). Groups were compared using a Student’s *t*-test or one-way analysis of variance (ANOVA), followed by Dunn’s post hoc test. Unless otherwise indicated, comparisons between groups were made using Student’s *t* test; *p* < 0.05 was considered significant.

## 5. Conclusions

The results of this study are significant in providing confirmation concerning the role of EtOH-TRPM7 actions in BMVECs that offers insight into the modulatory effects of EtOH at the BBB in the presence of HIV viral proteins. These findings have delineated the involvement of TRPM7 in EtOH concentration-dependent effects at the BBB which can be important in developing therapeutic strategies for persons living with HIV (PLWH) with alcohol abuse leading to BBB dysfunction. With the use of human endothelial cells, our findings have an added translational value in clinically caring for persons living with HIV who are abusing alcohol. 

## Figures and Tables

**Table 1:** TRP gene expression levels in mouse BMVECs.

Subfamily	Gene symbol (RefSeq #)	Relative Expression (2^−ΔΔCt^)
TRP ankyrin subfamily	*Trpa1* (NM_177781)	n.d. ^1^
TRP canonical subfamily	*Trpc1* (NM_011643)	3.22 × 10^−4^
	*Trpc2* (NM_011644)	6.23 × 10^−4^
	*Trpc3* (NM_019510)	n.d. ^1^
	*Trpc4* (NM_016984)	n.d. ^1^
	*Trpc5* (NM_009428)	9.35 × 10^−5^
	*Trpc6* (NM_013838)	n.d. ^1^
	*Trpc7* (NM_012035)	9.90 × 10^−5^
TRP melastatin subfamily	*Trpm1* (NM_001039104)	n.d. ^1^
	*Trpm2* (NM_138301)	4.36 × 10^−5^
	*Trpm3* (NM_177341)	n.d. ^1^
	*Trpm4* (NM_175130)	3.50 × 10^−3^
	*Trpm5* (NM_020277)	2.82 × 10^−4^
	*Trpm6* (NM_153417)	n.d. ^1^
	*Trpm7* (NM_021450)	3.48 × 10^−2^
	*Trpm8* (NM_134252)	n.d. ^1^
TRP vanilloid subfamily	*Trpv1* (NM_001001445)	n.d. ^1^
	*Trpv2* (NM_011706)	1.50 × 10^−4^
	*Trpv3* (NM_145099)	n.d. ^1^
	*Trpv4* (NM_022017)	1.52 × 10^−4^
	*Trpv5* (NM_001007572)	n.d. ^1^
	*Trpv6* (NM_022413)	n.d. ^1^
TRP mucolipin subfamily	*Trpml1* (NM_053177)	1.89 × 10^−2^
	*Trpml2* (NM_026656)	1.14 × 10^−3^
	*Trpml3* (NM_134160)	3.46 × 10^−5^
TRP polycystin subfamily	*Trpp1* (NM_013628)	1.89 × 10^−2^
	*Trpp2* (NM_008861)	1.14 × 10^−3^
	*Trpp3* (NM_181422)	3.46 × 10^−5^
	*Trpp5* (NM_016927)	2.77 × 10^−4^

^1^ n.d. = not detected (Ct ≥ 35).

## Data Availability

Not applicable.
